# Effects of caffeic acid phenethyl ester and melatonin on distraction osteogenesis: an experimental study

**DOI:** 10.1186/2193-1801-3-8

**Published:** 2014-01-03

**Authors:** Mehmet Erdem, Deniz Gulabi, Cengiz Sen, Seyit Ahmet Sahin, Ergun Bozdag

**Affiliations:** Orthopaedic and Traumatology Department, Faculty of Medicine, Sakarya University, Sakarya, Turkey; Orthopaedic and Traumatology Clinique, Dr. Lutfi Kirdar Kartal Training and Research Hospital, Semsi Denizer Cad. E-5 Yanyol, Cevizli Sapagi, 34890 Kartal, Istanbul, Turkey; Orthopaedic and Traumatology Department, Faculty of Medicine, Istanbul University, Istanbul, Turkey; Orthopaedic and Traumatology Clinique, Erbaa State Hospital, Tokat, Turkey; Mechanical Engineering Department, Istanbul Technical Faculty, Istanbul, Turkey

**Keywords:** Distraction osteogenesis, Extremity, Melatonin, Caffeic acid phenethyl ester

## Abstract

**Aim:**

The aim of this experimental animal model study is to investigate the effects of caffeic acid phenethyl ester (CAPE) and melatonin on the maturation of newly-formed regenerated bone in distraction osteogenesis.

**Methods:**

Unilateral femoral lengthening(extension) was applied to 39 adult male Wistar albino rats, which were randomly allocated to 3 groups of 13; control, melatonin and CAPE groups. Through a 7-day latent waiting period and 15 days of distraction, melatonin of 25 mg/kg and CAPE of 10 μmol/kg were administered to the respective groups. The animals were sacrificed on Day 82. Radiographic, histological and biomechanical evaluations were made and measurements were taken.

**Results:**

At the end of 82 days, the distraction osteogenesis area was seen to be completely filled with new bone formation in all 3 groups both radiologically and histologically. Biomechanically, the maximum torsional fracture strength (Maximum Torque (N-m)) of the melatonin group was higher compared to that of the control group, although it was not statistically significant (p > 0.05). The maximum torsional momentum of the CAPE group was statistically significantly high (p < 0.05). The degree of rigidity (N-m/deg) of both the melatonin and CAPE groups was higher than that of the control group and the CAPE group was found to be statistically significantly higher than the melatonin group (p < 0.05).

**Conclusion:**

Melatonin and CAPE increase the maturation of new bone in distraction osteogenesis. These effects are probably due to the reducing effect on bone resorption by inhibiting NF-κB and free oxygen radicals.

## Introduction

Distraction osteogenesis, which was popularised by Ilizarov (Ilizarov and Ledyaev 1992), is nowadays successfully used in many orthopaedic reconstructive surgical procedures. It is used in the treatment of chronic orthopaedic problems such as congenital and developmental limb length discrepancy, deformities, post-traumatic segmental bone loss, pseudoarthrosis and osteomyelitis successfully (Ilizarov and Ledyaev 1992; Alam et al. 2009; Sen et al. 2007). Distraction osteogenesis has three stages, the longest and most problematic of which is the consolidation stage. In this stage, the lengthy duration of the fixator restricts movement of the joint and increases the risk of pin site infection. Various methods have been recommended to reduce the external fixator time (EFT) and the external fixator index (EFI) and these have been reported with success in literature (Alam et al. 2009; Catagni et al. 2011; Emara et al. 2011; Lovisetti and Sala 2013). These may be listed as the use of autogenous bone graft, the application of ultrasonography (USG), electromagnetic waves, bone morphogenic protein, compression-distraction of the regenerative area, biphosphanate, calcitonin and platelet-rich plasma (PRP).

Melatonin is a hormone secreted by the pineal gland which regulates the biorhythm. It is known as antioxidant, and has the property of removing free oxygen radicals. In-vitro studies have shown melatonin to have a positive effect on osteoblastic differentiation and bone healing (Roth et al. 1999). In a study using rat tibia, Halici et al. ( 2010) showed melatonin to have an accelerating effect on bone healing. In an in vitro study, Nakade et al. ( 1999) showed melatonin stimulates proliferation and type 1 collagen synthesis in human bone cells, and suggesting that melatonin may act to stimulate bone formation. In an another study, Koyama et al. showed melatonin causes an inhibition of bone resorption and increase in bone mass by decreasing the RANKL- mediated osteoclast formation (Koyama et al. 2002).

Cafffeic acid phenethyl ester (CAPE) is one of the constituents of propolis, which is produced by honeybees. Various in vitro studies have shown this to have antiseptic, antibacterial, anti-inflammatory, immunomodulator, antioxidant and antimutagenic effects (Elmali et al. 2002). Our experimental investigation aimed to compare the effects of melatonin and CAPE with a control group in an animal model of distraction osteogenesis.

## Materials and method

Approval for the study was granted by Gaziosmanpaşa University Medical Faculty Animal Experiments Ethics Committee and the study was supported by Gaziosmanpaşa University Scientific Research Projects Commission. The study comprised 39 (it is not consistent with the number given in the first page (33))adult male Wistar albino rats with a mean weight of 384 gr (350–400). The rats were randomly allocated to 3 groups of 13, as Group 1, Melatonin group, Group 2, CAPE group and Group 3, control group.

**Figure 1:**
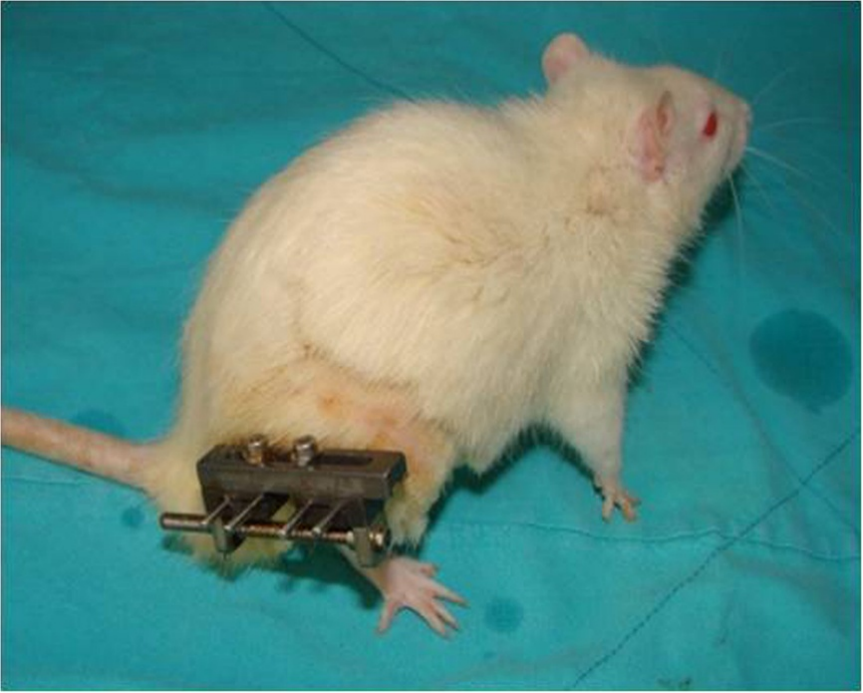
**The image of an albino rat, after the osteotomy and unilater external fixator application.**

Under sterile operating room conditions, general anaesthesia was administered intramuscularly as Ketamine (90 mg/kg) and xylazine (10 mg/kg). As a prophylaxis cefazoline sodium (20 mg/kg/day i.m.) was injected preoperatively, and eight hours postoperatively. The right lower extremity was shaved and prepared with povidone-iodine solution. An incision was made anterolaterally along the thigh. The fascia was cut longitudinally and with an approach between the quadriceps femoris and the hamstrings, the femur was exposed. Without separating the hamstring muscles from femoral periosteum, the quadriceps muscle was retracted medially. After opening a hole in the bone with a 1 mm K-wire, 4 × 1.5 mm self-taping screws were manually placed. A mini unilateral external fixator was placed over the self-taping screws. A mid-diaphysis bone osteotomy was made under continuous irrigation with a micro-oscillating saw and then the wound layers were closed. Intramuscular analgesics were administered for postoperative pain control. After a seven day waiting period, distraction was applied to each femur on a Schedule of 2 × 0,175 mm/day for 15 days to a total of 5 mm distraction (Figure 1). The animals were randomly allocated to three groups of 13 in each group as melatonin, CAPE and the control group. Throughout the latent and distraction phase (22 days), the following were administered to the groups intraperitoneally; to Group 1, melatonin (Sigma, St. Louis, MO, USA) (25 mg/kg), to Group 2, CAPE (Sigma, St. Louis, MO, USA) (10 μmol/kg), and to Group 3, the control group, physiological saline solution. Melatonin was dissolved in ethanol and further dilutions were made in saline. The final concentration of the ethanol was 1%. CAPE was dissolved in ethanol and further dilutions were made in saline. The final concentration of the ethanol was 1%. The control vehicle group was administrated 1% alcohol in saline (5 ml/kg/day) intraperitoneally during experiment. At the end of fifteen days distraction was terminated. On the 82. day, the experiment was terminated by sacrificing the all rats in each group via an intravenous administration of a fatal dose of sodium pentobarbital. For histological evaluation, extremities dissected from their soft tissues were fixed in 10% buffered formaldehyde solution for 48 h, and then treated with a rapid decalcification solution for 12 h. After the decalcification procedure, femurs were sampled longitudinally from distracted segments. For each femoral segment, four cuts were taken from the distraction area and routine histological preparation was carried out. After routine histological preparation, samples were embedded in paraffin. From paraffin blocks sections of 4 μm in width were taken and stained with hematoxylin-eosin. Sections stained with hematoxylin-eosin were examined under light microscope by a pathologist who was unaware of the allocation of rats into groups. Radiographic evaluation of new bone formation in the distraction area was made according to Lane and Sandhu ( 1987) criteria (Table 1). Histological evaluation of the samples were made according to Huddlestone et al. histological grading system (Huddleston et al. 2000) (Table 2).

**Table 1:** **Radiological evaluation system (Lane and Sandhu** 1987 **)**

Bone formation	Points
Lack of bone formation	0
Bone formation filling 25% of the defect	1
Bone formation filling 50% of the defect	2
Bone formation filling 75%of the defect	3
Bone formation filling 100%of the defect	4
Union	
No union	0
Onset of union	1
Complete radiological union	2
Remodeling	
None	0
Formation of intramedullary channel	1
Formation of cortex	2
Maximal total score	10
Bone formation	4
proximal union	2
Distal union	2
Remodeling	2

**Table 2:** **Histological Evaluation System (Huddleston et al.** 2000 **)**

Points	Histological findings
1	Fibrous tissue
2	Predominantly fibrous tissue with some cartilage
3	Equal amounts of fibrous and cartilage tissue
4	All cartilage
5	Predominantly cartilage tissue with some woven bone
6	Equal amounts of cartilage and woven bone
7	Predominantly woven bone with some cartilage
8	Entirely woven bone
9	Woven bone and some mature bone
10	Lamellar(mature) bone

**Figure 2:**
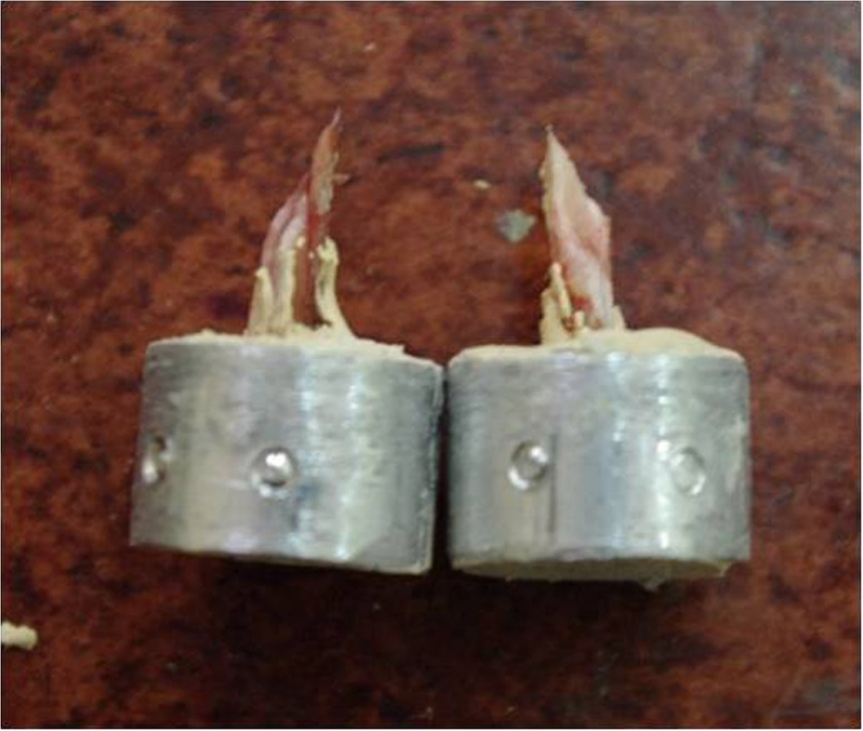
**The image of the biomechanical test machine.**

For biomechanical tests, femurs of each of the three groups of rats were fixed to cylindrical aluminium pipes with adhesive paste (steel paste). They were fixed to metal cylindrical blocks and prepared for the test by the upper sides being held in the jaws of a universal test machine (MTS 858 Bionix II, MN, USA, 55344) and the lower sides being attached to a load cell (MTS Axial-Torsional Load Transducer, (2500 N/25 N-m), MN USA, 55344) which would measure axial and torsional loads. After the samples were attached to the test machine, each sample was exposed to torsional loading at 50°/min angular speed. The tests were continued until damage was seen on the samples. Applying the same test scenario to each sample, the time, torsional degree and torsional momentum value simultaneously at 10Hz frequency, were recorded (Figure 2). Using Microsoft Excel program on the collected data, the following values were obtained at the moment of damage for each sample during torsional loading which leads to damage; axial maximum torsional breaking load (N-m), torsional breaking degree. Rigidity was calculated (N-m/deg). In evaluating the radiographic and histological data obtained from the study, SPSS(Statistical Package for Social Science) for Windows 15.0 programme was used for statistical analysis, Kruskal Wallis test on the continuous variables was used in the study for radiologic and histological results. Significance was evaluated according to values p < 0.05.

One way variance analysis (ANOVA) was used in the comparison between the 3 groups when normal distribution was shown according to the Kolmogorov Smirnov normality test on the continuous variables used in the study. In paired comparisons between the groups, the Sheffe and Tamhane tests were used according to the variance homogeneity status. Continuous variables were stated as arithmetical mean and standard deviation (SD). A p value of <0.05 was accepted as statistically significant. Calculations were made using a statistical software package (PASW v18, SPSS Inc, Chicago, IL).

## Results

**Figure 3:**
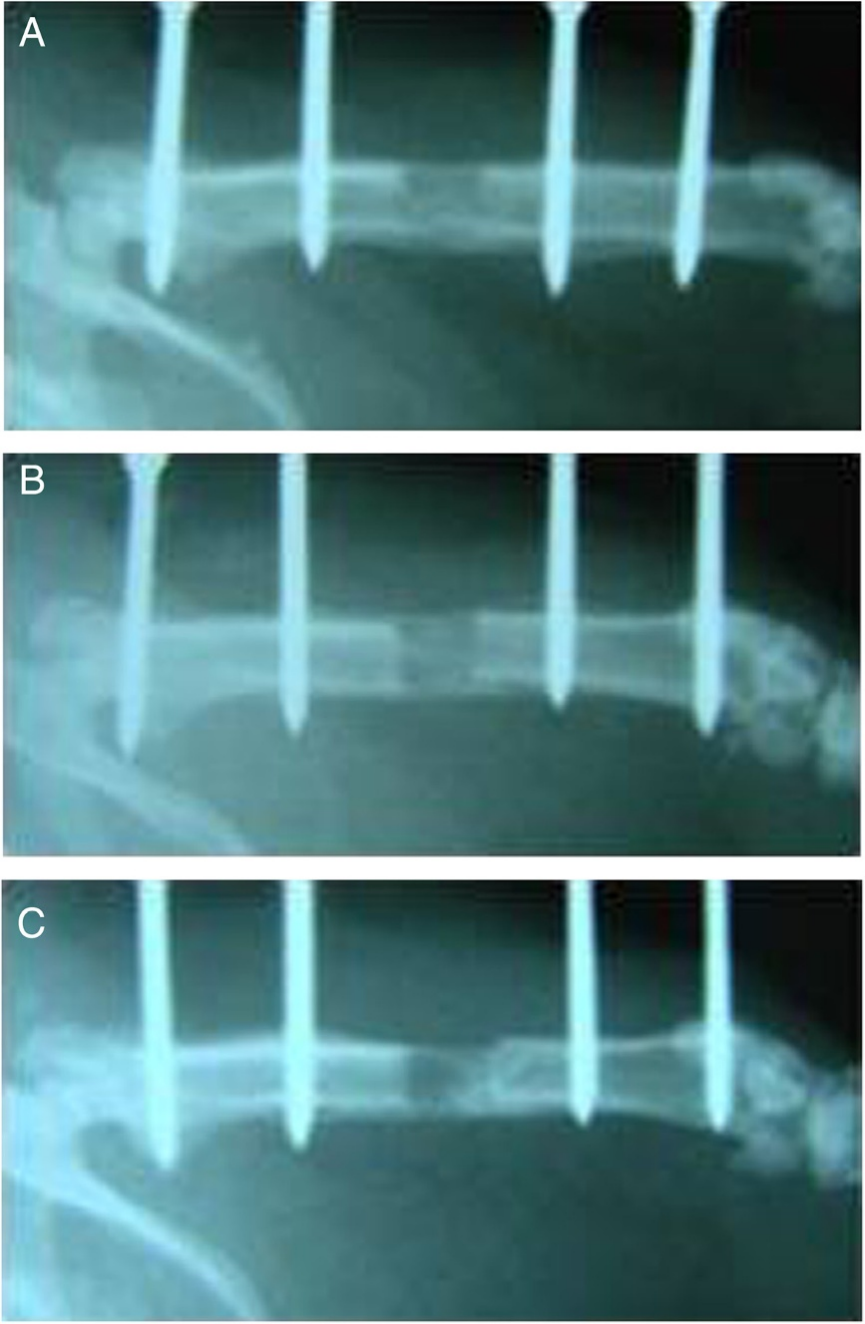
**The radiographic evaluation of the femurs at the end of 82 days. A**: The regeneration of the distraction part consisted of bone formation, proximal and distal union, and remodeling. The maximal total score was 10. **B**: CAPE group: The regeneration of the distraction part showed bone formation, proximal and distal union, and remodelling at the end of 82 days. The maximal total score was 10. **C**: Control group: The regeneration of the distraction part showed bone formation, proximal and distal union, but limited remodeling. The maximal total score was 9.

**Figure 4:**
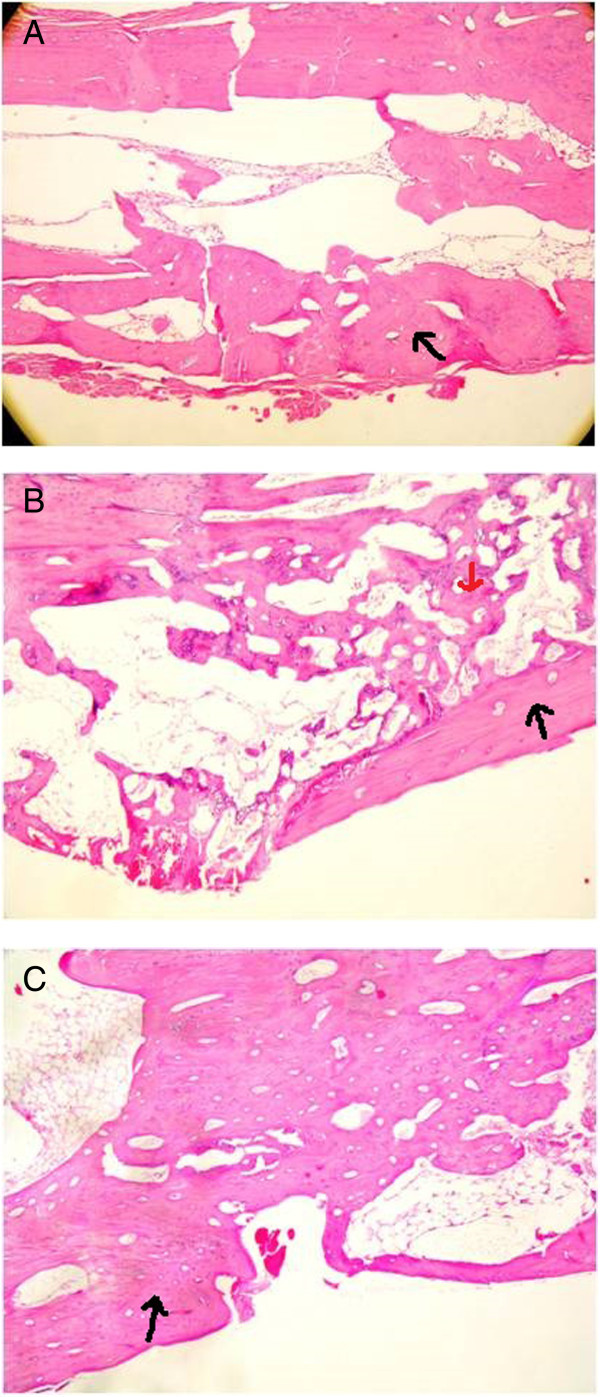
**The samples were embedded in paraffin.** From paraffin blocks sections of 4 μm in width were taken and stained with hematoxylin-eosin, and were examined under light microscope (HE × 20). **A**: Melatonin group: The callus formation consisted of mostly well-developed trabecular bone (Black arrow). Haversian canals were seen in the section. Chondroid tissue areas were observed limitedly in the callus at the end of 82 days. (HE,×20). **B**: CAPE group: Callus was formed by predominantly trabecullar bone(Black arrow), and chondroid tissues(red arrow) were rarely observed in the callus at the end of 82 days. (HE,×20). **C**: Control group: Callus formation consisting of equal amounts of trabecular bone and chondroid tissue in the control(Black arrow) at the end of 82 days. (HE, ×20).

The study was completed with 33 rats due to the death of two rats in group 1, two in group II, and two in group III due to unexpected death, infection, and diarrhea. Histological and radiological results obtained at the end of 82.days were not statistically significant, but we observed better regeneration in groups 1 and 2 compared with group 3. The mean radiological score for group 1 was 9.18 ± 0.6, 9.09 ± 0.54 for group 2, and 8.64 ± 0.5 for the control group (Figure 3A-C). The mean histological score for group 1 was 9.09 ± 0.3, 9.0 ± 0.44 for group 2, and 8.82 ± 0.4 for the control group. During the histopathological examination, significant osteoblastic activity in the distraction area, trabecular bone formation were observed. Trabecular bone tissue was the dominant component in callus tissue of the group 1 and 2, whereas cartilaginous tissue was apparent in group 3. And also we detected significant osteoblastic activity and apparent callus formation in group 1 and 2 compared to group 3. There is not a statistically significant results among the groups (p > 0.05) (Table 3) (Figure 4A-C).

**Table 3:** **Mean radiological and histological scores of the groups at the end of 82 days**

		Mean ± SS (median)	p
**Radiological score**	**Melatonin**	9,18 ± 0,60 (9)	**0,065**
	**CAPE**	9,09 ± 0,54 (9)	
	**Control**	8,64 ± 0,50 (9)	
**Histological score**	**Melatonin**	9,09 ± 0,30 (9)	**0,254**
	**CAPE**	9,00 ± 0,44 (9)	
	**Control**	8,82 ± 0,40 (9)	

Kruskal Wallis test.

**Figure 5:**
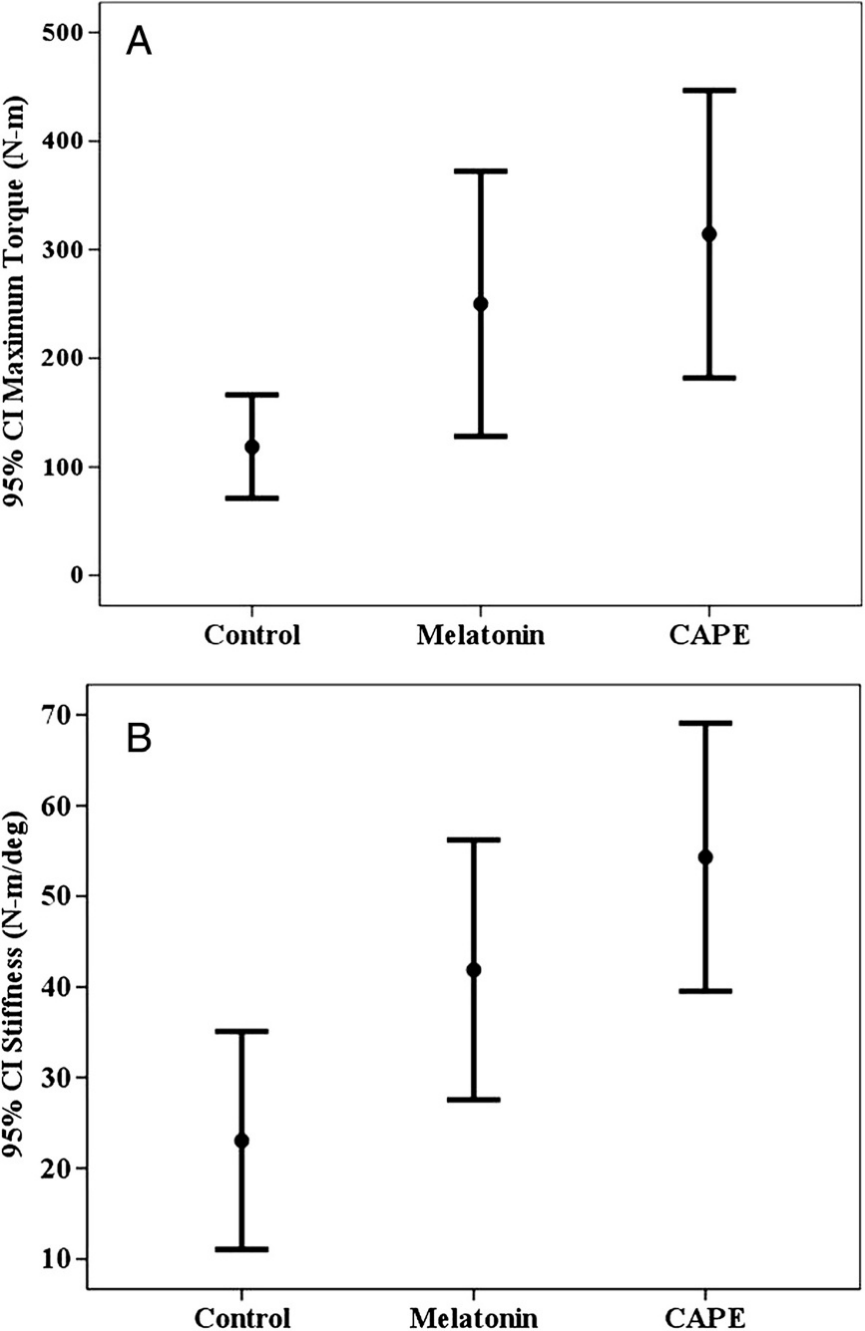
**Fracture strength and rigidity level of the groups. A**: Maximum fracture strength of the groups. CAPE had the maximum fracture strength. Maximum fracture strength of the melatonin group was greater than control group. **B**: Level of rigidity of the groups. The rigidity of the CAPE group was greater than Melatonin group, the rigitidy of the melatonin group was greater than control group.

When the biomechanic test results were examined, the maximum torsional fracture momentum (MFM) (maximum torque (N-m)) which caused fracture of the sample was greater in the melatonin group than the control group although the difference was not statistically significant (p > 0.05). This value was greater in the CAPE group than the control group and was determined to be statistically significant (p < 0.05). The CAPE group value was greater than that of the melatonin group but was not statistically significant (p > 0.05) (Figure 5A). The degree of rigidity in the groups were determined as melatonin > control group, CAPE > control group and CAPE > melatonin group (p < 0.05) (Figure 5B).

## Discussion

The concept of distraction osteogenesis, as described by Codivalla ( 1994) and developed by Ilizarov (Ilizarov and Ledyaev 1992) opened a new era in orthopaedic reconstructive surgery. Problematic orthopaedic cases such as congenital or developmental extremity shortness, deformities, pseudoarthrosis, extremity reconstruction following bone tumour resection and osteomyelitis started to be treated with this method (Ilizarov and Ledyaev 1992; Alam et al. 2009; Sen et al. 2007).

There are three phases to distraction osteogenesis (Emara et al. 2011). The first stage as the latent phase starts with an osteotomy and lasts for mean 7–10 days. In the second stage as the distraction stage, lengthening is made with a unilateral or circular external fixator applied to the proximal and distal bone fragments of the osteotomy. In the third and final stage as the consolidation stage, the regeneration, formed during the lengthening matures. The longest of these stages is the consolidation stage. It is the stage of maturation of the regeneration formed between the two ends of the osteotomy line during distraction. Extension of this stage creates patient dissatisfaction and intolerance. In addition, that it can cause various complications such as pin site infection, fixator loosening and stiffness in neighbouring joints has been reported in literature. Several authors have proposed various methods to reduce external fixator duration at this stage of the procedure and have reported successful results. Compression-distraction on the fixator, grafting with autogenous bone graft, the use of bone morphogenic protein, PRP, biphosphanate and calcitonin are the most prominent methods (Alam et al. 2009; Emara et al. 2011; Lovisetti and Sala 2013).

The histopathology of bone formation in the distraction stage was elucidated by Aronson ( 1997). The bone formation between the ends of the osteotomy, which is named regeneration, occurs by intramembranous bone formation, as after the osteotomy, firstly type 1 collagen fibres are formed then these collagen fibres are aligned paralel to the lengthening vector and start to cluster on the transverse axis. At this stage, chondroblasts start to appear and cartilage production and bone ossification on this starts. At the end of the distraction phase, osteon canals and osteoblastic activity are seen between the bone ends and mineralisation starts. In the consolidation stage, according to the Wolf principle, this regeneration area remodels to bone tissue as the intramedullary canal and the sharp cortex surrounding it (Emara et al. 2011). The duration of this regeneration formation and the duration of its maturation are defined by several factors. The age of the patient, diabetes, the use of immunosuppressive medication, cigarette smoking, blood circulation of the osteotomy side and external or internal stability of the bone are important factors (Catagni et al. 2011; Emara et al. 2011). Therefore, young, healthy, albino rats were selected for this study. The osteotomies were made with a low-speed motor without excessive periosteal dissection as Kojimoto et al. ( 1988) reported that protecting the periosteum rather then the endosteum of the bone had a more positive effective on callus formation. Following the recommended 7-day latent phase (Ilizarov and Ledyaev 1992), lengthening of 2 × 0.175 mm was started. From the radiographs taken in the 82^nd^day, the lengthening rhythm was seen to be sufficient.

Various treatment protocols have been recommended by several authors for maturation in the consolidation stage of the regeneration which is formed in the distraction stage. Yamane et al. ( 1999) showed that ED-71, which is a Vitamin D product, enhanced thickening in the callus tissue after the distraction stage. Mizumoto et al. ( 2003) demonstrated an increase in bone mineral density at the distraction osteogenesis stage with the use of rh-BMP. In a study by Li et al. (Yamane et al. 1999), thrombin peptide 508 (TP508) was seen to contribute to the bone maturation and consolidation phases of distraction-osteogenesis. Sen et al. ( 2006) reported positive effects of calcitonin on distracted segment consolidation. Shimazaki et al. ( 2000) demonstrated that low-dose USG had a positive effect on maturation. In an experimental study by Sen et al. ( 2007), NSAIDs were seen to have a negative effect on maturation.

In experimental studies, the formation of free oxygen radicals (FOR) have been seen in the reperfusion stage following temporary ischaemia in the inflammation stage of bone healing (Erdem et al. 2010). FOR, especially superoxide anion are a mediator in the formation of osteoclasts and have a significant effect in bone resorption (Garett et al. 1990) Göktürk et al. ( 1995) showed the negative effects of FOR on bone healing. We did not encounter any studies on the effects of CAPE on the outcome of distraction osteogenesis. Caffeic acid phenethyl ester (CAPE) is one of the molecular agents of propolis, which is produced by honeybees, and is known to have anti-inflammatory, anticarcinogenic, immunomodulatory and antioxidant properties (Lovisetti and Sala 2013; Nakade et al. 1999). In recent years, it has started to be used frequently in medical treatments because of these properties. Elmali et al. ( 2002) reported that the effect of CAPE on osteoarthritis in an experimental rabbit model was shown by inhibiting nuclear factor kapa B (NF-κB) which leads to cartilage damage. In another experimental animal model, Ang et al. ( 2009) showed that CAPE inhibited osteoclast differentiation and activation by inhibiting NF-κB associated with RANKL. In the current study, positive effect of CAPE were seen in the distraction-osteogenesis consolidation stage. This is thought to be due to making the FOR ineffective and inhibiting NF-κB which plays a key role in the differentiation and activation of osteoclasts. This effect was demonstrated in the statistically high values of the biomechanical breaking strength and rigidity of the newly-formed bone tissue compared to those of the control group (p < 0.005).

Melatonin is secreted by the pineal gland. It has a regulatory effect on body heat and circadian and seasonal rhythms. Melatonin is a significant free oxygen radical scavenger (Erdem et al. 2010; Cuzzocrea and Reiter 2001; Satomura et al. 2007). There is a positive effect of melatonin on bone formation. This effect was shown as an increase in osteoblastic activity by Roth et al. ( 1999). In the current study, the rates of biomechanical breaking and level of bone rigidity were found to be high in the melatonin group compared to the control group. When the biomechanical breaking point and rigidity value of the CAPE group were compared with the melatonin group, although the values were high, no statistical significance was determined.

In an experimental study, Clafshenkel et al. ( 2012) recommended that, bone defects implanted with CA-Melatonin scaffolds show greatest degree of bone remodelling both quantitatively and qualitatively. The histological analysis of the samples clarified the osteoid tissue mineralization and bone formation. The addition of PRP with CA did not show the same improvement as in the case of Melatonin. Shimazaki et al. ( 2000) concluded that ultrasound can accelerate bone maturation in distraction osteogenesis in rabbits, even in states of poor callotasis. Histological analysis showed no tissue damage attributed to ultrasound exposure. Lesalchot et al. ( 2011) investigated the influence of rh-BMP-2 on the consolidation phase in a distraction osteogenesis model. Qualitative radiographic evaluation revealed hypertrophic calluses. Densitometric analysis revealed the bone mineral content (BMC) was significantly higher in the rh-BMP-2 treated animals.

Li et al. ( 2005) stated that recombinant human bone morphogenetic protein- 2 (rhBMP-2) reinforced the consolidation phase of distraction osteogenesis in rabbit models. To compare the effects of melatonin and CAPE with USG, rhBMP-2 and PRP, further experimental and human studies should be studied.

## Conclusion

The use of CAPE and melatonin can be beneficial in the maturation of the newly-formed regeneration and reducing the external fixator time and index in distraction osteogenesis and due to the biomechanical torsional breaking strength and high rigidity rate, will reduce the possibility of refracture and angulation which may be seen following fixator removal.
